# Maternal fish and shellfish intake and pregnancy outcomes: A prospective cohort study in Brittany, France

**DOI:** 10.1186/1476-069X-6-33

**Published:** 2007-10-24

**Authors:** Laurence Guldner, Christine Monfort, Florence Rouget, Ronan Garlantezec, Sylvaine Cordier

**Affiliations:** 1 Inserm U625, GERHM, IFR140, Campus de Beaulieu, Rennes, F-35042 France; Univ-Rennes I, Rennes, F-35042 France; 2 National School of Public Health (ENSP), avenue du Professeur Léon Bernard, CS 74312, 35043 Rennes CEDEX, France; 3 Medical network on perinatality "Bien naître en Ille-et-Vilaine", Aile de direction – Hôtel-Dieu CHU, 2 rue de l'Hôtel-Dieu, CS 26419, 35064 Rennes CEDEX, France

## Abstract

**Background:**

Recommendations about risks and benefits of seafood intake during pregnancy have been published in the last decade, but the specific health effects of the different categories of seafood remain unknown. Fish and shellfish may differ according to their fatty acid content and their concentration of chemical pollutants and toxins. Not taking these particularities into account may result in underestimating of both the positive and negative effects of seafood on birth outcomes and partly explains inconsistent results on the subject.

**Methods:**

In the PELAGIE cohort study, including 2398 pregnant women from Brittany, we fit multiple linear and logistic regression models to examine associations of fish (salt-water fish only) and shellfish intake before pregnancy with length of gestation, birthweight, and risks of preterm births, low birthweight or small-for-gestational-age (SGA) babies.

**Results:**

When fish and shellfish consumptions were considered simultaneously, we observed a decrease in the risk of SGA birth with increasing frequency of fish intake: OR = 0.57 (95%CI: 0.31 to 1.05) for women eating fish twice a week or more compared with those eating it less than once a month. The risk of SGA birth was significantly higher among women eating shellfish twice a week or more than among those eating it less than once a month: OR = 2.14 (95%CI: 1.13 to 4.07). Each additional monthly meal including fish was significantly related to an increase in gestational length of 0.02 week (95%CI: 0.002 to 0.035). No association was observed with birthweight or preterm birth.

**Conclusion:**

These results suggest that different categories of seafood may be differently associated with birth outcomes, fish consumption with increased length of gestation and shellfish consumption with decreased fetal growth.

## Background

Seafood is a major source of long chain polyunsaturated fatty acids (PUFA). Reviews of the health benefits of n-3 FA on pregnancy outcome by Allen in 2001 [1] and Facchinetti in 2005 [2] suggest they have a beneficial effect on gestational length and fetal growth. Some observational studies [3,4] and clinical trials in the general population [5,6] and in women with high-risk pregnancies [7,8] show that n-3 PUFA, fish oil and seafood are associated with prolonged length of gestation or decreased preterm-birth (PB) rates [3-8], increased birthweight [4,5,9,10], and a reduced proportion of small-for-gestational age (SGA) births [11]. Results are nonetheless inconsistent: some clinical trials and studies find seafood consumption is not associated with birthweight [6,12], fetal growth [8,13] or length of gestation [10-15], while others suggest it has a negative effect on fetal growth [15,16]. This inconsistency has been widely discussed and explained in part by the possibility that the beneficial effects of eating seafood during pregnancy may be balanced by the potential adverse effects of seafood contaminants such as polychlorinated biphenyls (PCBs), dioxins, or other polychlorinated compounds and metals [17-19]. A recent study of Danish women showed that consumption of fatty fish, which are more likely than lean fish to contain persistent organic pollutants, is associated with reduced fetal growth [20].

Despite the many studies on this topic in populations in Denmark, the Faroe Islands, England and the US, this association has never been studied in France. Brittany is an interesting site for such a study: not only is seafood consumption high, but consumption of shellfish is sufficiently high to allow for a separate analysis of the effects of fish and shellfish. The studies mentioned above analyzed relations between pregnancy outcome and fish (+/- whale) consumption alone [9,16,20] or global seafood consumption [3,4,10,15], without distinguishing between fish and shellfish intake. Because the levels of contaminants and fatty acids in these two categories may differ, their health effects may differ as well, and specific profiles of seafood consumers may be at higher risk for adverse pregnancy outcome.

Accordingly we investigated the relation of seafood – that is, fish and shellfish – intake before pregnancy to length of gestation, birthweight and decreased fetal growth in a cohort of 2398 pregnant women in Brittany.

## Methods

### Population and study design

The aim of the PELAGIE cohort was to study the role of environmental pollutants on pregnancy course and outcome and on children's health and development. Women in two districts (Ille et Vilaine and Finistere) of Brittany (France) were enrolled from April 2002 to February 2005 during the first trimester of their pregnancy, when they saw participating gynecologists or ultrasonographers for prenatal care. They were then followed through delivery. Participants provided informed consent for data collection, and the appropriate ethics committees approved the study procedures. At inclusion, women completed a questionnaire that asked about social and demographic characteristics of the family, obstetric history, occupational activity, nutrition and lifestyle. Medical information about pregnancy, delivery, and the newborn's health was obtained from midwives, pediatricians and hospital medical records.

In all, 2398 women returned the inclusion questionnaire (for an estimated participation rate of 80%), and delivery information was available for 2353 (98%). This analysis excludes the stillbirths (n = 28) and multiple pregnancies (n = 24).

### Dietary assessment

The questionnaire administered at enrollment (during the first trimester of pregnancy), included food frequency questions that inquired about the usual prevalence of consumption, before pregnancy, of 18 specific categories of food, originally selected because of their contribution to polychlorinated dibenzo-dioxin/furan (PCDD/PCDF) intake in the French population [21]. Seafood consumption was examined by 4 items about intake of "salt-water fish (including salmon)", "mollusks (oysters, mussels, etc.), "large crustaceans (crabs, spider crabs, etc.)" and "small crustaceans (shrimp, etc.)". Women with data missing for any of these 4 items (n = 23) were excluded. More detailed information about fish or shellfish species, freshwater fish intake, or portion sizes was not available in this study. The 5 categories of frequency ranged from "never or less than once a month" to "every day" and were translated into 0.5, 3, 10, 20 and 30 meals of the specific food each month. Because of small cell sizes, the initial five categories of fish consumption frequency were recoded as three, that is, "never or less than once a month", "1–4 a month" and "2 or more a week". The total number of shellfish meals (that is, mollusks and crustaceans) was computed and then categorized as "never or less than once a month", "1–4 a month" and "2 or more a week".

### Outcome assessment

Birthweight (in g) was abstracted from hospital records. Length of gestation (in weeks) was assessed by the midwife or the gynecologist at the time of delivery and took into account the date of last menstrual period (recorded at enrollment) and ultrasound examination during the first trimester of pregnancy (between 10 and 14 weeks of amenorrhea, in accordance with mandatory routine practice in France). Discrepancies were resolved by clinical judgment. Preterm birth was defined as length of gestation < 37 weeks and low birthweight (LBW) as birthweight < 2500 g. Newborns classified as small-for-gestational age (SGA) were defined by a birthweight lower than the 10th percentile of birthweight distribution for a given gestational age and sex, according to French reference curves [22].

### Covariates and potential confounders

Smoking habits at inclusion were used to define smoking status and level of consumption at the end of the first trimester. Maternal height and pre-pregnancy weight were used to compute maternal body mass index (BMI, kg/m^2^) before pregnancy. The list of covariates for the multivariate models included maternal age (<25, 25–29, 30–34, ≥ 35 years), marital status (single, couple), educational level (≤ baccalaureate examination, 2 years and 3 years or more of education after high school), primiparous (yes, no), maternal height (<160, 160–<165, 165–<170, ≥170 cm), maternal BMI (<18.5, 18.5–<25, 25–<30, ≥30 kg/m^2^), level of smoking at inclusion (non-smoker, <10 cigarettes per day, ≥ 10 per day), alcohol consumption (< or ≥ 1 drink per week), sex of the child and diabetes (before pregnancy or pregnancy-induced). Length of gestation was also included for birthweight and LBW analyses.

### Statistical analysis

Statistical significance was assessed with the chi-square statistic and with trend tests for all categorical variables and analysis of variance for continuous variables. The relation between fish or shellfish consumption and birth outcomes was assessed with multiple linear regression for length of gestation or birthweight and with multiple logistic regression for categorical variables (PB, LBW and SGA). Covariates for regression analyses included those mentioned above. For the LBW analysis, length of gestation was categorized as <35, 35–36, 37, 38, 39 and ≥ 40 weeks. Tests for trend in ORs for fish and shellfish intake included these 2 variables in multivariate models coded as 0, 1, and 2 for the three levels of intake frequency [3]. Statistical analyses were performed with SAS software version 9.1 (SAS Institute, Inc., Cary, North Carolina).

## Results

Mean maternal age at inclusion was 30.2 (standard deviation SD = 4.1) years; 65% of the women had 2 years or more of education after high school and 46% were primiparous (see Table 1). Mean BMI was 22.2 kg/m^2 ^(SD = 3.6): 11.8% of the women were overweight (between 25 and 30 kg/m^2^), and 4.1% were considered obese (30 kg/m^2 ^or more). Of the 11.3% (n = 256) who reported smoking at inclusion, only 58 smoked 10 cigarettes/day or more. Mean birthweight was 3324 g (SD = 484) for girls and 3448 g (SD = 498) for boys, and mean length of gestation was 39.4 weeks (SD = 1.6). In our population sample, 4.2% of births were classified as preterm, 3.1% as LBW and 5.3% as SGA. Risk of preterm birth was highest when parity was lowest and when the mother had diabetes. LBW rates tended to decrease as parity increased and to increase with smoking and drinking (i.e., among women drinking one drink or more per week during pregnancy). The rates of SGA babies were higher among primiparous smokers and among women who had not passed baccalaureate than among their better educated peers; SGA rates also decreased with increasing maternal height or BMI.

The total number of seafood meals per month averaged 7.6, including 4.6 meals of fish, and 3.0 of shellfish (table 2). Assuming a mean portion size of 135 g for fish and 200 g for shellfish (data from the French SU.VI.MAX study [23]), this corresponds to a mean daily intake of about 20.4 g/day of fish and 19.7 g/day of shellfish. About one quarter of the women (26.6%, n = 606) reported eating fish at least twice a week. Most women reported eating shellfish (mollusks, large and small crustaceans) less than once a month (respectively 81%, 91% and 74%). Fish and shellfish intake were significantly correlated (correlation coefficient = 0.25 p < 10^-4^). Individual factors associated with increased seafood consumption, such as increasing maternal age and educational level, were similar for fish and shellfish. Heavy smokers ate fish but not shellfish less frequently than the other subjects.

**Table 1:** Description of study population and variations of adverse pregnancy outcome rates

		**PB rates N = 94 (4.2%)**		**LBW rates N = 71 (3.1%)**		**SGA rates N = 120 (5.3%)**	
	**N (%)**	**%**	**p**	**%**	**p**	**%**	**p**

**Maternal age**							
<25	214 (9.4)	6.5	0.3	3.7	0.6	5.6	0.9
25–29	945 (41.5)	3.8		2.9		5.0	
30–34	808 (35.5)	4.0		3.6		5.2	
≥35	310 (13.6)	3.9		2.3		6.2	
**Marital status**							
Couple	2226 (97.8)	4.2	1.0	3.1	0.7	5.2	0.1
Single	50 (2.2)	4.1		4.0		10.0	
**Education level**							
None/primary education	16 (0.7)	6.3	0.8	6.3	0.6	12.5	0.2
Secondary education	360 (15.9)	4.8		4.2		7.2	
Baccalaureate	410 (18.1)	4.4		2.7		4.2	
2 years higher education	592 (26.1)	3.9		3.0		5.1	
3 years or more higher education	890 (39.2)	3.9		2.9		4.9	
**Parity**							
0	1045 (46.0)	5.2	0.02	4.2	0.004	7.4	<10^-4^
1	831 (36.6)	2.8		2.7		4.0	
≥2	396 (17.4)	4.1		1.0		2.5	
**Maternal height (cm)**							
<160	421 (18.6)	5.5	0.3	4.5	0.3	9.3	0.0003
160 – <165	793 (34.9)	4.1		3.2		4.9	
165 – <170	617 (27.2)	4.3		2.4		4.7	
≥170	437 (19.3)	3.0		2.8		3.0	
**Pre-pregnancy BMI (kg/m^2^)**							
<18.5	180 (7.9)	5.6	0.3	3.3	0.8	3.9	0.04
18.5–<25	1725 (76.2)	3.7		3.3		6.0	
25–<30	267 (11.8)	5.7		2.3		1.9	
≥30	93 (4.1)	5.4		2.2		5.4	
**Nb of cig/day smoked at inclusion**							
0	2022 (88.8)	4.3	0.7	2.9	0.006	4.9	0.02
<10	198 (8.7)	3.1		3.0		7.6	
>= 10	58 (2.5)	3.5		10.3		12.1	
**Alcohol drinking**							
0 drink per week	2216 (98.3)	4.1	0.3	3.0	0.009	5.1	0.2
1 drink per week or more	39 (1.7)	7.7		10.3		10.3	
**Diabetes**							
No	2200 (96.6)	3.9	0.006	3.1	0.7	5.2	0.6
Yes	78 (3.4)	10.3		3.9		6.4	

**Table 2:** Frequency of seafood consumption according to category

**Frequency of consumption n (%)**	**Fish**	**Mollusks**	**Large crustaceans**	**Small crustaceans**	**Total shellfish intake**
Never or less than once a month	406 (17.8)	1846 (81.0)	2068 (90.8)	1683 (73.9)	1548 (68.0)
1 to 4 a month	1266 (55.6)	415 (18.2)	203 (8.9)	572 (25.1)	548 (24.1)
2 or 3 times a week	555 (24.4)	17 (0.8)	7 (0.3)	21 (0.9)	165 (7.2)
4 to 6 times a week	49 (2.1)	0	0	1 (0.05)	16 (0.7)
Every day	2 (0.1)	0	0	1 (0.05)	1 (0.04)

Mean number of meals a month (sd)	4.6 (4.1)	1.0 (1.2)	0.8 (0.9)	1.2 (1.6)	3.0 (3.0)

Table 3 reports the frequency of adverse pregnancy outcomes and the odds ratios (OR) and 95% confidence intervals (CI) for PB and for LBW and SGA newborns according to frequency of fish and shellfish intake, both when these were included separately (models 1 for fish and 2 for shellfish) or simultaneously (model 3) in regression models. The results for models 1 and 2 show nonsignificant inverse associations between fish intake and risks of PB, LBW and SGA, between shellfish intake and PB risk, and a nonsignificant positive association between shellfish intake and risk of LBW or SGA newborns. After mutual adjustment for both categories of seafood intake (model 3), associations between fish and shellfish intake and risk of SGA newborns were stronger and the positive association between risk of SGA babies and shellfish intake was significant (p = 0.02). The risk of SGA birth was 1.33 (95%CI: 0.83 to 2.11) and 2.14 (95%CI: 1.13 to 4.07) times higher respectively for women eating shellfish 1 to 4 times per month and those eating it 2 times a week or more, compared with those eating fish less than once a month. An analysis of subcategories of shellfish intake (i.e. mollusks, small and large crustaceans) showed that the latter association was mostly explained by intake of large crustaceans: the risk of SGA babies was twice as high among women who ate large crustaceans more than once a month (p = 0.03) compared with those eating this type of seafood once a month or less.

**Table 3:** Odds ratios of PB, LBW and SGA, according to frequency of seafood intake

	**Frequency of adverse outcomes (%)**			**Models 1 and 2^a,c ^OR (95%CI)**			**Model 3^b,c ^OR (95%CI)**		
	**PB**	**LBW**	**SGA**	**PB**	**LBW^d^**	**SGA**	**PB**	**LBW^d^**	**SGA**

**Frequency of fish intake**				*Model 1*					
<1 time a month *n = 406*	4.5	4.4	7.1	1	1	1	1	1	1
1–4 times a month *n = 1266*	4.6	3.3	5.1	1.01 (0.58–1.78)	0.62 (0.24–1.61)	0.79 (0.48–1.29)	1.06 (0.60–1.87)	0.59 (0.23–1.55)	0.72 (0.44–1.19)
≥2 times a week *n = 606*	3.0	1.8	4.5	0.65 (0.32–1.32)	0.65 (0.21–2.09)	0.68 (0.37–1.22)	0.71 (0.35–1.46)	0.59 (0.18–1.91)	0.57 (0.31–1.05)
p (linear trend)	0.1	0.008	0.04	0.2	0.5	0.2	0.3	0.4	0.07
**Frequency of shellfish intake**				*Model 2*					
<1 time a month *n = 1548*	4.6	3.4	4.8	1	1	1	1	1	1
1–4 times a month *n = 548*	3.3	2.4	5.7	0.77 (0.45–1.32)	1.06 (0.44–2.52)	1.21 (0.77–1.91)	0.81 (0.47–1.39)	1.09 (0.45–2.62)	1.33 (0.83–2.11)
≥2 times a week *n = 182*	2.8	3.3	7.7	0.62 (0.24–1.57)	2.10 (0.67–6.59)	1.89 (1.01–3.52)	0.66 (0.26–1.70)	2.24 (0.70–7.15)	2.14 (1.13–4.07)
p (linear trend)	0.06	0.3	0.05	0.2	0.3	0.05	0.3	0.2	0.02

PB: Preterm Birth; LBW: Low Birthweight; SGA: Small for Gestational Age; BMI: Body Mass Index; p: degree of significance

^a ^adjusted models including either fish (model 1) OR shellfish (model 2) consumption. ^b ^adjusted model including both fish AND shellfish consumption. ^c ^adjusted for maternal age, marital status, education level, parity, bmi, height, smoking status, alcohol consumption, diabetes and sex of the child. ^d^adjusted for duration of gestation

In order to account for the possible residual effect of gestation length on SGA risk estimates, we re-ran complete regression models that included this variable, but this did not make any difference in the results.

When we studied continuous outcome variables, there was a trend toward increasing length of gestation with increasing fish consumption, with a small but statistically significant increase (p = 0.03) of 0.02 week (0.002–0.035) in the duration of gestation for each additional fish meal per month (table 4). No association was observed between fish or shellfish consumption and birthweight. Because it has been suggested that smoking might act as a modifying factor [10], we also stratified our analyses by smoking status, but this did not change our results.

**Table 4:** Results of linear regression for pregnancy duration and birthweight according to seafood intake

	**Length of gestation (weeks)**				**Birthweight (grams)**			
	**Simple linear regression**		**Multiple linear regression^b^**		**Simple linear regression^c^**		**Multiple linear regression^b,c^**	
	
	**β (95%CI)**	**p**	**β (95%CI)**	**p**	**β (95%CI)**	**p**	**β (95%CI)**	**p**

**Frequency of fish intake^a^**	0.021 (0.004–0.037)	0.01	0.018 (0.002–0.035)	0.03	0.777 (-3.446–5.000)	0.7	-1.556 (-5.587–2.476)	0.8
**Frequency of shellfish intake^a^**	-0.014 (-0.036–0.009)	0.2	-0.018 (-0.041–0.005)	0.1	1.000 (-4.796–6.795)	0.7	1.50 (-4.017–7.016)	1.0

^a ^models included both fish and shellfish for mutual adjustment. ^b ^adjusted for maternal age, marital status, education level, parity, bmi, height, smoking status, alcohol consumption, diabetes and sex of the child. ^c^adjusted for duration of gestation

## Discussion

We investigated the role of seafood intake before pregnancy and analyzed the influence of the two principal categories of seafood on pregnancy outcome. This study showed a small statistically significant increase in length of gestation with increasing fish intake of uncertain clinical relevance and a significantly increased risk of SGA births with increasing shellfish intake, mostly explained by intake of large crustaceans. When we fit models with continuous outcomes, we found no evidence of a relation between seafood intake and birthweight. The rates of adverse outcomes in our population (4.2% for PB, 3.1% for LBW and 5.3% for SGA newborns) were relatively low compared with national data. In 2003, the national perinatal survey [24] estimated French rates of PB and LBW among singleton live births at 5.0% and 5.5%, respectively. The high rate of ascertainment of pregnancy outcomes (98%) and the good quality of medical data obtained lead us to consider that these rates are not underestimated in our study population and reflect regional specificity. However, we cannot exclude the fact that these low rates may also be due in part to enrollment bias, if the women included were likely to be healthier. SGA was defined according to standard national reference curves for birthweight, published in 1996. The SGA birth rate was lower than 10%, however, as observed in other populations recently with the same reference curves [25]. Length of gestation was assessed according to information in the medical files, and all women began prenatal care during the first trimester. We thus assume that this information is accurate. Measuring pregnancy duration in days instead of weeks would, however, have provided more sensitive and precise information about the increase observed.

Most determinants of birthweight and gestational length were taken into account, except weight gain during pregnancy. This variable was measured in a subgroup of our sample and did not appear to add information in the models. Our population had a particularly high educational level and a particularly low smoking rate, but other major known risk factors nonetheless significantly affected gestational age, birthweight and other outcomes.

Our conclusions can only be suggestive, however, because of some limitations in dietary assessment and the absence of biomarkers. Because we had no detailed information about the different types of fish consumed (including oily vs. white fish), we could not estimate either FA or contaminant intake. Food frequency was assessed by a single questionnaire, administered during the first trimester and asking about habitual fish intake just before pregnancy. We therefore could not take into account possible changes during pregnancy. However, a recent study conducted among Danish women [3] showed similar decreases in duration of gestation among women never consuming fish compared to those consuming fish at least once a week, when fish intake was assessed either during the first or the second trimester of pregnancy. Seafood consumption was high in our population, especially shellfish, compared with national data, with mean intake estimated at about 20.4 g/day of fish and 19.7 g/day of shellfish. A national diet survey conducted in 1998–1999 estimated intake for these foods among women at 28 g/day for fish and 4 g/day for shellfish [26], that is, the same order of magnitude for fish, but much lower for shellfish than in our population, due to regional characteristics.

### Comparison with other studies

#### Length of gestation

Our results are concordant with several observational studies and clinical trials that show positive associations between prolonged length of gestation (or decreased risk of preterm birth) and fish intake levels [3,4], n-3 fatty acid concentration in cord serum phospholipids or maternal erythrocytes as biomarkers of marine n-3 FA intake [16,27], or fish oil supplementation [5,8]. In our study, the association between gestational length and fish intake corresponded to a mean decrease of 0.52 day (95%CI: 0.05 to 0.99) among woman eating fish less than once a month, compared to those eating fish 4 times a month, and 1.03 day (95%CI: 0.10 to 1.97) when compared to women eating fish twice a week. These estimates are low compared with recent results published by Olsen and colleagues [3], who found a mean gestation length 3.91 days (95%CI: 2.24 to 5.58) shorter among women never consuming fish compared to those eating it at least once a week, as assessed during the first trimester of pregnancy. This difference may be partly explained by the inclusion of women eating fish less than once a month in our nonconsumer category. Another possible explanation is that our estimates concern pre-pregnancy fish intake, differing from other studies that studied usual fish consumption during pregnancy.

#### Birthweight, Small for Gestational Age

Studies suggesting that fish intake has a positive effect on birthweight include one clinical trial [5] and observational studies [4,9,10]. This positive relation exists even when no association is observed with length of gestation [9,10]. A study published in 2004 by Rogers et al showed an inverse association between fish intake and the risk of intra-uterine growth retardation after excluding mothers who ate shellfish [11]. In contrast, one clinical trial and two cohort studies reported finding no relation between fish or fish oil intake and fetal growth [8,13] or birthweight [12]. A recent study [15] showed a negative relation between n-3 polyunsaturated FA intake and fetal growth, but it did not present risk estimates according to different categories of seafood. An inverse association between eicosapentaenoic acid (EPA) cord serum concentration and birthweight adjusted for gestational age was also shown among Faroese women [16], but this study did not investigate shellfish intake.

The trend in our crude analyses between increasing fish intake and both decreased risk of LBW (p = 0.008) and SGA (p = 0.04) was considerably attenuated when we adjusted for confounders, particularly length of gestation for LBW and tobacco consumption for both outcomes.

When we looked at total seafood consumption, we found no association with risk of SGA births; the ORs for the intermediate and highest categories of total seafood intake were respectively 0.97 (0.54–1.76) and 1.02 (0.65–1.58), p = 0.9, compared with the lowest category. The trend towards a negative relation between risk of SGA birth and fish intake may then have been balanced by the positive trend for shellfish intake. This justifies separate analyses and mutual adjustment for these two categories of seafood.

Our observations are consistent with the hypothesis of that n-3 FA (higher in fish than shellfish) may have a positive impact that may be counterbalanced in part by the deleterious impact of some contaminants, such as metals and organochlorines (often high in shellfish due to their filter feeding characteristics). The French Food Safety Agency published national data about the seafoods most commonly consumed according to level of consumption, about fish and shellfish contents in n-3 PUFA, and their concentration in trace elements and pollutants [28]. The data for the subsample of women of childbearing age (18–44 years) living in Lorient (south Brittany) indicate that levels of n-3 PUFA in the most frequently consumed fish were quite similar to those in the most frequently consumed large crustaceans. The large crustaceans, however, contained more dioxins (including PCDD, PCDF and PCBs "dioxin like") and more other PCBs than fish. Concentrations of metals (especially arsenic and cadmium) were also higher in large crustaceans than in fish.

## Conclusion

In a population with low background rates of adverse pregnancy outcomes and high seafood consumption, we observed that different categories of seafood had different associations with birth outcomes. Our findings suggest that increased fish consumption is associated with increased length of gestation and suggest that high shellfish intake is associated with an increased risk of SGA births. Further investigation should elucidate the respective effects of the different categories of seafood based upon measurements of toxics and beneficial nutrients. Levels of contaminants in some categories of seafood (especially shellfish) should be monitored because heavy consumers may be at particular risk.

## Competing interests

The author(s) declare that they have no competing interests.

## Authors' contributions

LG was involved in statistical analysis, interpretation of the results, and drafted the manuscript. CM assisted in the planning of the study, data collection, data management and analysis. FR was the pediatrician coordinator and was involved in study planning and medical data collection. RG was in charge of medical data collection, coding and quality control. SC conceived and designed the study, supervised the overall project and was involved in the interpretation of the results and revision of the paper. All authors read and approved the final manuscript.
